# Evaluation of Efficacy of Adjuvant Radiotherapy in Well-Differentiated Liposarcoma Patients with Positive Surgical Margins: A Population-based Study

**DOI:** 10.1155/2022/5735679

**Published:** 2022-09-08

**Authors:** Haoran Wang, Boran Chen, Ying Cen, Junjie Chen

**Affiliations:** ^1^Department of Burn and Plastic Surgery, West China Hospital, Sichuan University, Chengdu 61000, China; ^2^Department of Neurosurgery, West China Hospital, Sichuan University, Chengdu 61000, China

## Abstract

**Background:**

The use of adjuvant radiotherapy (RT) for well-differentiated liposarcoma (WD-LPS) patients with positive surgical margins is unclear. We aim to compare the overall survival (OS) and cancer-specific survival (CSS) of well-differentiated liposarcoma patients with positive surgical margins in an RT group and non-RT group.

**Methods:**

WD-LPS patients with positive margins from 2000 to 2018 were extracted from the Surveillance, Epidemiology, and End Results (SEER) database and divided into two groups: RT group and non-RT group. Kaplan–Meier survival analysis with the log-rank test was performed to evaluate the difference of overall survival (OS) and cancer-specific survival (CSS) between groups. Univariate and multivariate Cox proportional hazard analyses were performed to identify important prognostic factors of OS and CSS. Analyses were adjusted using propensity-score matching.

**Results:**

We identified 2549 patients: 377 (14.79%) with RT and 2172 (85.21%) without RT. The median age was 61 years, and the median follow-up time was 68 months. The log-rank test revealed that there was no significant difference of CSS between RT and non-RT groups (*P* = 0.81). The 5-year and 10-year CSS were comparable (*P* = 0.418 and *P* = 0.987). Additionally, the use of RT was neither an independent prognostic factor for OS nor CSS. Age, sex, tumor site, the use of chemotherapy were independent prognostic factors for OS after propensity score matching, while race and the tumor site were independent prognostic factors for CSS.

**Conclusion:**

Adjuvant RT had no significant improvement on OS and CSS of WD-LPS patients with positive surgical margins.

## 1. Introduction

Liposarcoma cases account for about 25% of all soft tissue sarcoma (STS) cases. According to the World Health Organization (WHO) latest classification, liposarcoma can be divided into five subtypes: (i) atypical lipomatous tumor (ALT) or well-differentiated liposarcoma (WD-LPS), (ii) dedifferentiated liposarcoma (DD-LPS), (iii) myxoid liposarcoma, (iv) pleomorphic liposarcoma, and (v) myxoid pleomorphic liposarcoma [1]. WD-LPS represents about 40–45% of all liposarcoma cases, typically presents in older adults, and tends to arise in deep soft tissue of the limbs and retroperitoneum [2]. General trends and characteristics of the clinical behavior of ALT/WD-LPS had been described in prior studies, and they are low-grade tumors, minimally symptomatic and slowly growing [3]. However, if WD-LPS patients are improperly handled in the early clinical stage and tumor recurs repeatedly, it may transform into DD-LPS. WD-LPS has no metastatic potential unless it transforms into DD-LPS, which is a nonlipomatous tumor [4]. DD-LPS has a more aggressive behavior compared to WD-LPS and has a high possibility of local recurrence reported to be about 85% [5]. Besides, the metastatic rate of DD-LPS was reported around 14%, and metastatic tumors have a 5-year disease-specific survival rate of around 5% [6]. The primary treatment for WD-LPS is surgical resection. Because of the increased latency, the tumors often have much more chance to grow to a large size before diagnosis; thus, it is difficult to achieve R0 resection. Prior results showed that patients who underwent incomplete gross resection had significantly shorter overall survival (OS) than with R0 resection [7]. However, there are no studies focusing on the role of adjuvant RT on WD-LPS patients with positive surgical margins specifically.

Based on the data extracted from the Surveillance, Epidemiology, and End Results (SEER) database, this study aimed to explore whether adjuvant RT improves the OS and cancer-specific survival (CSS) of WD-LPS patients with positive surgical margins and identify independent prognostic factors of OS and CSS. We present the following article in accordance with the STROBE reporting checklist.

## 2. Methods

### 2.1. Study Population

Data were derived from the SEER 18 registry database, which consists of cancer registries from 18 geographic areas and covers approximately 28% of the United States population, from 2000 to 2018 using SEER^*∗*^Stat (version 8.3.9, https://seer.cancer.gov/seerstat/).

We initially identified all the WD-LPS patients with the following inclusion criteria: (1) patients with the International Classification of Diseases for Oncology, Third Edition (ICD-O-3) histology code of 8851/3; (2) patients aged ≥18 years; (3) patients who underwent surgery and with positive surgical margins, i.e., with subtotal resection of tumors. The exclusion criteria were as follows: (1) patients with incomplete record of age, sex, tumor size; histologic subtype, RT, chemotherapy, cause of death, or follow-up time; (2) patients with a follow-up time of 2 months or less (in order to account for immortal time bias); (3) patients with a prior malignancy diagnosis; (4) patients without histological confirmation of diagnosis; (5) patients who received RT prior to surgery or intraoperative RT.

### 2.2. Variables

The variables including age at diagnosis (≤60 years or >60 years), year of diagnosis (2000–2009 or 2010–2018), sex, race (white, black, or other), primary tumor site (retroperitoneum, extremity, or other), tumor size (<5 cm, 5–10 cm or ≥10 cm), chemotherapy data, and RT data were obtained. The cutoff values of age at diagnosis (60 years) and tumor size (<5 cm, 5–10 cm or ≥10 cm) were determined based on former publications about extremity liposarcomas, which were found to be independent risk factors of decreased OS and CSS [8]. Patients with the race of American Indian/Alaska Native and Asian/Pacific Islander were categorized to “another” group. Patients who underwent RT after surgery were assigned to the RT group, while those without RT were assigned to the non-RT group. The outcome of interest of this study was OS and CSS. OS was defined as the interval from the date of the primary diagnosis to the date of death due to any cause. CSS was defined as the interval from the date of the primary diagnosis to the date of liposarcoma-specific death.

### 2.3. Statistical Analysis

Characteristics of patients were compared between the RT group and the non-RT group using *χ*^2^ tests. OS and CSS between groups were compared using the Kaplan–Meier method with log-rank tests. The univariable and multivariable Cox proportional hazard models were used to identify contributors to OS and CSS and to calculate the hazard ratio (HR) and the corresponding 95% confidence interval (CI).

Additionally, propensity score matching was performed to adjust confounding factors. Age, year of diagnosis, sex, race, tumor size, tumor site, and use of chemotherapy were used for matching. Patients were matched with the one-to-one nearest-neighbor method without replacement. The Cox proportional hazards regression model was performed in the matched cohorts to determine signiﬁcant contributors to OS and CSS. A *P* value less than 0.05 was considered statistically significant. All the statistical analyses were conducted using Stata (version 15.1, Stata Corp., College Station, TX, USA) and *R* program (version 3.6.3).

## 3. Results

### 3.1. Demographic and Clinical Characteristics of WD-LPS Patients with Positive Surgical Margins

**Figure 1:**
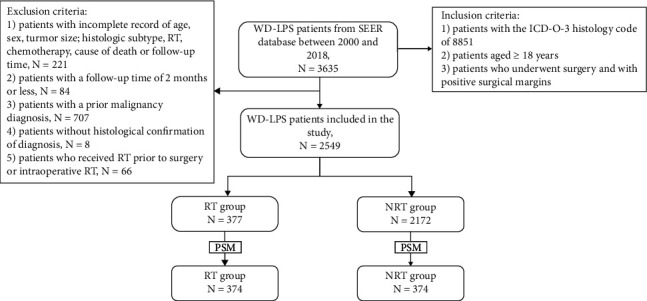
The flow diagram of patient selection and grouping of this study. WD-LPS, well-differentiated liposarcoma; SEER, Surveillance, Epidemiology, and End Results; ICD-O-3, International Classification of Diseases for Oncology, Third Edition; RT, radiotherapy; NRT, nonradiotherapy.

Data for a total of 2549 patients were included in this study according to the inclusion and exclusion criteria (Figure 1). The baseline demographics and clinical characteristics are listed in Table 1. The median age and follow-up time of all patients were 61 years and 68 months, respectively. There was male predominance (59.4%) in the whole cohort. More patients were diagnosed after 2010 (58.7%). As for race, whites made up the majority (79.8%). More than half of the tumors were located at the trunk or extremities (53.9%), while head and neck tumors were the least (2.7%). Only a few patients received chemotherapy (1.1%). There were 377 patients in the RT group and 2172 patients in the non-RT group. Baseline characteristics were comparable between the RT group and the non-RT group in terms of age, sex, race, primary tumor site, tumor size, and chemotherapy.

### 3.2. Analysis of Variables Associated with OS and CSS among Patients in RT and Non-RT Groups

#### 3.2.1. Overall Survival

**Figure 2:**
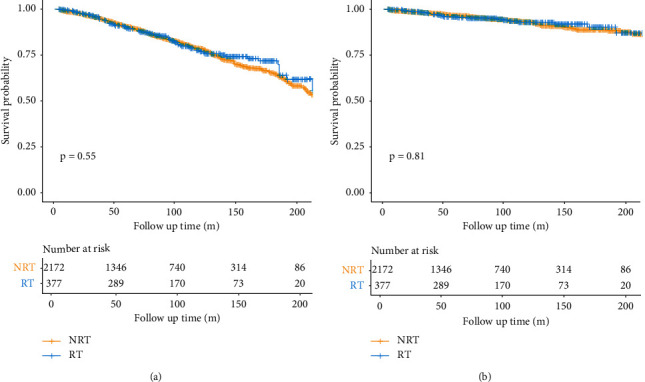
Kaplan–Meier curves of OS (a) and CSS (b) comparing the radiotherapy group and the nonradiotherapy group before propensity score matching.

Kaplan–Meier curves for OS in RT and non-RT groups are depicted in Figure 2(a). In Kaplan–Meier analysis, RT was not significantly associated with better OS (*P*=0.55). In addition, the 5-year OS and 10-year OS of the whole cohort in RT and non-RT groups are depicted in Supplementary Table 1. Our study revealed that RT was not significantly associated with better 5-year (*P*=0.578) and 10-year (*P*=0.632) OS in WD-LPS patients with positive surgical margins. The results of univariate analyses are shown in Supplementary Table 2. At multivariable analysis, older patients' age (*P* < 0.001), male (*P* < 0.001), primary tumor sites located at the trunk and extremities (*P* < 0.001), visceral organs (*P*=0.004), and received chemotherapy (*P* < 0.001) were all negative independent prognostic factors for OS (Supplementary Table 2). However, adjuvant RT was not an independent prognostic factor for OS (*P*=0.549).

#### 3.2.2. Cancer-Specific Survival

Kaplan–Meier curves for CSS in RT and non-RT groups are depicted in Figure 2(b). In Kaplan–Meier analysis, adjuvant RT was not significantly associated with better CSS (*P*=0.81). In addition, the 5-year CSS and 10-year CSS of the whole cohort in RT and non-RT groups are shown in Supplementary Table 1. Our study revealed that adjuvant RT was not significantly associated with better 5-year (*P*=0.418) and 10-year (*P*=0.987) CSS in WD-LPS patients with positive surgical margins. The results of univariate analyses are also shown in Supplementary Table 3. At multivariable analysis, older patients' age (*P*=0.001), male (*P*=0.004), other races (*P*=0.024), primary tumor sites located at the trunk and extremities (*P* < 0.001), visceral organs (*P*=0.002), tumor size ≥10 cm (*P*=0.028), and received chemotherapy (*P* < 0.001) were all significantly associated with worse CSS (Supplementary Table 3). However, adjuvant RT was not an independent prognostic factor for CSS (*P*=0.811).

#### 3.2.3. Propensity Score Analysis of Adjuvant RT and Prognostic Factors

The propensity score-matched dataset included 748 patients. After propensity score matching, the difference in demographic and clinical characteristics of the RT and non-RT group patients disappeared. The comparison of the p value before and after propensity score matching is depicted in Table 1.

**Figure 3:**
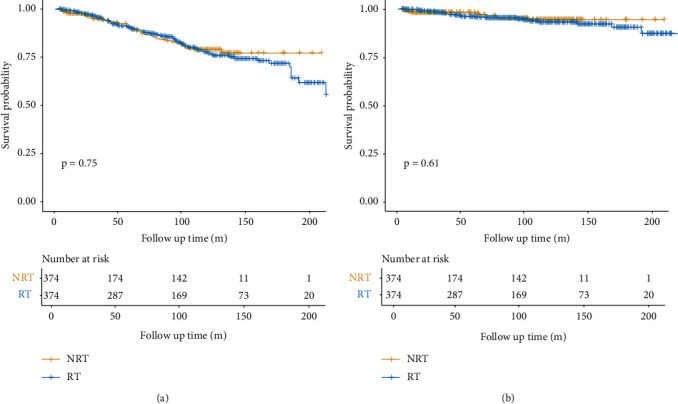
Kaplan–Meier curves of OS (a) and CSS (b) comparing the radiotherapy group and the nonradiotherapy group after propensity score matching.

After propensity score matching, the Kaplan–Meier curves for OS and CSS in RT and non-RT groups are shown in Figure 3. Patients treated with adjuvant RT still showed no better 5-year (*P*=0.964) and 10-year (*P*=0.507) OS than patients who did not receive adjuvant RT, the same for 5-year (*P*=0.192) and 10-year (*P*=0.540) CSS (Supplementary Table 4). After the adjustment by propensity score matching, the results of multivariable analysis for the independent prognostic factors for CSS were different but OS was the same as before (Tables 2 and 3). For the independent prognostic factors for CSS, age, sex, the year of diagnosis, tumor size, and received chemotherapy were no longer significantly associated with CSS. Only patients from other races (*P*=0.022) and primary tumor sites located at the trunk and extremities (*P* < 0.001) were significantly associated with worse CSS. On the other hand, adjuvant RT was still not an independent prognostic factor for both OS (*P*=0.753) and CSS (*P*=0.608) after propensity score matching.

## 4. Discussion

In this series of 2549 WD-LPS patients with positive surgical margins in an 18-year time span, there was no significant difference in Kaplan–Meier curves for OS in RT and non-RT groups, the same for CSS. In addition, the analysis showed that adjuvant RT was not significantly associated with better 5-year and 10-year OS and CSS. Similarly, when 748 patients were selected in a propensity score-matched analysis, patients treated with adjuvant RT still showed no significant benefit in 5-year and 10-year OS and CSS.

### 4.1. Effects of Adjuvant RT

Previous studies focused mainly on the effects of adjuvant RT on the OS outcome and rarely for the CSS outcome analysis. Some of the previous studies found similar results. A large series of ATL/WD-LPS by Cassier et al. showed that there was no significant difference in OS between the RT group and the non-RT group in ATL/WD-LPS patients including positive and negative margin (*P*=0.105) [9]. In addition, the results of a study of 607 localized retroperitoneal WD-LPS or dedifferentiated liposarcoma patients who underwent surgical resection (including macroscopically complete surgical resection and positive microscopic margins) with or without RT in 8 high-volume sarcoma centers showed that the RT effect on OS was not statistically significant after inverse probability of treatment weighting adjustment [10]. Besides, another large series of over 3752 primary extremity soft tissue sarcoma patients (containing WD-LPS patients with positive surgical margins) by Callegaro et al. showed that RT was not associated with OS [11]. However, several studies on retroperitoneal sarcoma (containing WD-LPS patients with positive surgical margins) hold opposite results. In a European pooled analysis by Roeder et al., intraoperative electron radiation therapy combined with external beam radiation therapy (EBRT) after limb-sparing surgery resulted in encouraging OS in extremity soft tissue sarcoma patients [12]. In addition, Roeder et al. found that although addition of EBRT did not result in improved OS, there was a significant survival benefit for patients treated with preoperative EBRT compared with no EBRT at all [13]. Besides, a Scandinavian study of 97 retroperitoneal sarcoma patients (including liposarcoma patients with positive surgical margin) reported that 5-year OS was 71% in the adjuvant RT group and 52% in surgery alone group (*P*=0.019), suggesting that adjuvant RT was significantly associated with an improved 5-year OS [14]. This difference may be due to the small number of cases, the diverse population characteristics, and the inclusion of other kinds of STS and positive/negative surgical margins in their study.

### 4.2. Prognostic Factors of CSS and OS

We also explored the independent prognostic factors of CSS and OS in WD-LPS patients with positive surgical margins with the adjustment by propensity score matching. After the adjustment, we identified only patients from other races (*P*=0.022) and primary tumor sites located at the trunk and extremities (*P* < 0.001) were significantly associated with worse CSS. Previous studies on the multivariate analysis associated with CSS had shown different results. Wang et al. found that age at diagnosis <60 was signiﬁcantly correlated with improved CSS [15]. In addition, a study by Ye et al. identified that age, sex, and tumor size were independent prognostic variables for CSS in extremity liposarcoma patients [16]. This difference may be due to the lack of propensity score matching in these studies avoiding the bias in analysis, and propensity score analysis showed a strong effect on removing bias and identifying truly useful prognostic factors. Besides, extremity liposarcoma is not a single entity, and different liposarcoma subtypes should be analyzed separately for their prognostic patterns and characteristics. The analysis of our study focused on WD-LPS patients with positive surgical margins, which may be more valuable and helpful for clinical decisions on this specific group of patients.

As for the multivariate analysis on OS, after the adjustment, we identified age (*P* < 0.001), sex (*P*=0.031), primary tumor location (*P* < 0.001), and received chemotherapy (*P*=0.033) were independent prognostic factors for OS in WD-LPS patients with positive surgical margins (Table 2). There was a high similarity between our results and previous results on significant prognostic factors of OS. A large study of liposarcoma (including WD-LPS patients with positive surgical margins) by Greto et al. found that age was a significant factor associated with OS, in which age >65 y was related with worse OS (*P*=0.0001) [17]. Toulmonde et al. reported male gender was an independent prognosis factor associated with poor OS in retroperitoneal sarcoma patients including WD-LPS [18]. In addition, for the prognosis of WD-LPS, a tumor location is of great importance [19]. According to former research results, different anatomical locations of WD-LPS had different risks of dedifferentiation, and retroperitoneal WD-LPS had a much higher risk of dedifferentiation than WD-LPS in the limbs (>20% in the retroperitoneum vs. <2% in the limbs) [20]. On the other hand, WD-LPS located at extremities/trunk wall/head-neck has a lower risk of metastasis [21]. These results supported our analysis on the primary site of a tumor as an independent prognostic factor of OS. Another previous study by Dario Callegaro holds opposite results on the prognostic values of chemotherapy, and their study included 1106 patients after propensity score matching; chemotherapy was not an independent prognostic factor for the OS (*P*=0.054) [11]. The reason causing this difference may be the patients they included in the study containing other subtypes of liposarcoma. In addition our results were more specific on WD-LPS patients with positive surgical margins only. Last but not least, our results revealed that adjuvant RT was not an independent prognostic factor for both OS and CSS after propensity score matching, which further proved the conclusion that adjuvant RT is not associated with better OS and CSS for WD-LPS patients with positive surgical margins.

### 4.3. Limitations

The limitations of this study are mainly the inherent limitations of the SEER database. First, WD-LPS is rare and difficult to diagnose. The inherent limitation of all SEER-based studies is that the laboratory technology of each registry included in SEER lacks central pathological review and uniformity, which may lead to potential differences in histological diagnosis. However, we tried to reduce the potential inaccuracy by including patients confirmed by histology. Another limitation is the lack of data on the RT dose and time. Moreover, the recurrence rate was not evaluated in this study because of the lack of this information in the SEER database. This limitation requires more detailed information on WD-LPS patients by collecting data from multiple tumor centers. Furthermore, this study was conducted within the United States population; thus, the results might not be generalizable to patients of other countries.

## 5. Conclusion

In conclusion, we found that adjuvant RT had no significant improvement on OS and CSS of patients with WD-LPS with positive surgical margins. Considering the adverse reactions and side effects of adjuvant RT, we do not recommend using adjuvant RT on patients with WD-LPS with positive surgical margins. In addition, our multivariable analysis with the adjustment of propensity score matching revealed that only patients from other races and primary tumor sites located at the trunk and extremities were significantly associated with worse CSS. Besides, we identified that age, sex, primary tumor location, and receipt of chemotherapy were independent prognostic factors for OS.

## Figures and Tables

**Table 1:** Characteristics of well-differentiated liposarcoma patients with positive surgical margins with or without adjuvant radiotherapy before and after propensity score matching.

Variables	Before PSM			After PSM		
	Nonradiotherapy (*n*, %)	Radiotherapy (*n*, %)	*P* value	Nonradiotherapy (*n*, %)	Radiotherapy (*n*, %)	*P* value
Number of patients	2172	377		374	374	
Age			1			0.884
≤60 years	1062 (48.9)	184 (48.8)		182 (48.7)	184 (49.2)	
>60 years	1110 (51.1)	193 (51.2)		192 (51.3)	190 (50.8)	

Sex			0.077			0.883
Male	1307 (60.2)	208 (55.2)		208 (55.6)	206 (55.1)	
Female	865 (39.8)	169 (44.8)		166 (44.4)	168 (44.9)	

Year of diagnosis			<0.001			0.942
2000–2009	862 (39.7)	190 (50.4)		189 (50.5)	188 (50.3)	
2010–2018	1310 (60.3)	187 (49.6)		185 (49.5)	186 (49.7)	

Race			0.682			1.000
White	1733 (79.8)	301 (79.8)		298 (79.7)	298 (79.7)	
Black	197 (9.1)	30 (8.0)		30 (8.0)	30 (8.0)	
Other	242 (11.1)	46 (12.2)		46 (12.3)	46 (12.3)	

Primary site			0.118			0.993
Retroperitoneum	286 (13.2)	34 (9.0)		32 (8.6)	34 (9.1)	
Head and neck	55 (2.5)	13 (3.4)		12 (3.2)	12 (3.2)	
Trunk and extremities	1163 (53.5)	212 (56.2)		215 (57.5)	212 (56.7)	
Visceral organs	668 (30.8)	118 (31.3)		115 (30.8)	116 (31.0)	

Tumor size			0.816			0.945
<5 cm	257 (11.8)	44 (11.7)		47 (12.6)	44 (11.8)	
5–10 cm	858 (39.5)	143 (37.9)		141 (37.7)	142 (38.0)	
≥10 cm	1057 (48.7)	190 (50.4)		186 (49.7)	188 (50.3)	

Chemotherapy			0.245			0.737
No	2150 (99.0)	370 (98.1)		370 (98.9)	369 (98.7)	
Yes	22 (1.0)	7 (1.9)		4 (1.1)	5 (1.3)	

**Table 2:** Univariate and multivariate Cox proportional hazard models for overall survival after propensity score matching.

Variable	Univariate analysis		Multivariate analysis	
	HR (95% CI)	*P* value	HR (95% CI)	*P* value
Age				
≤60 y	Reference		Reference	
>61 y	3.11 (2.08–4.65)	<0.001	3.29 (2.18–4.96)	<0.001

Sex				
Male	Reference		Reference	
Female	0.67 (0.46–0.98)	0.036	0.64 (0.44–0.96)	0.031

Year of diagnosis				
2000–2009	Reference			
2010–2018	1.10 (0.70–1.75)	0.677		

Race				
White	Reference			
Black	0.93 (0.48–1.79)	0.819		
Other	1.13 (0.66–1.92)	0.659		

Primary site				
Retroperitoneum	Reference		Reference	
Head and neck	0.99 (0.37–2.10)	0.780	0.79 (0.31–2.02)	0.624
Trunk and extremities	0.35 (0.21–0.60)	<0.001	0.30 (0.17–0.51)	<0.001
Visceral organs	0.68 (0.40–1.18)	0.174	0.54 (0.30–0.95)	0.033

Tumor size				
<5 cm	Reference		Reference	
5–10 cm	0.47 (0.27–0.83)	0.008	0.69 (0.39–1.24)	0.214
≥10 cm	0.86 (0.53–1.39)	0.537	1.13 (0.67–1.90)	0.655

Chemotherapy				
No	Reference		Reference	
Yes	5.60 (2.04–15.33)	0.001	6.10 (2.10–17.72)	0.001

Radiotherapy				
No	Reference			
Yes	1.06 (0.73–1.55)	0.753		

HR, hazard ratio; CI, confidence interval.

**Table 3:** Univariate and multivariate Cox proportional hazard models for cancer-specific survival after propensity score matching.

Variable	Univariate analysis		Multivariate analysis	
	HR (95% CI)	*P* value	HR (95% CI)	*P* value
Age				
≤60 y	Reference			
>61 y	1.44 (0.73–2.84)	0.291		

Sex				
Male	Reference			
Female	1.00 (0.51–1.96)	1.000		

Year of diagnosis				
2000–2009	Reference		Reference	
2010–2018	2.61 (1.21–5.62)	0.014	1.89 (0.84–4.24)	0.121

Race				
White	Reference		Reference	
Black	1.61 (0.55–4.73)	0.383	1.96 (0.65–5.85)	0.229
Other	2.36 (1.05–5.31)	0.037	2.61 (1.15–5.94)	0.022

Primary site				
Retroperitoneum	Reference		Reference	
Head and neck	0.27 (0.03–2.09)	0.208	0.36 (0.04–2.88)	0.334
Trunk and extremities	0.14 (0.05–0.34)	<0.001	0.14 (0.06–0.36)	<0.001
Visceral organs	0.53 (0.23–1.21)	0.131	0.51 (0.22–1.18)	0.115

Tumor size				
<5 cm	Reference			
5–10 cm	0.63 (0.18–2.15)	0.458		
≥10 cm	1.57 (0.54–4.53)	0.408		

Chemotherapy				
No	Reference		Reference	
Yes	8.34 (1.96–35.49)	0.004	3.89 (0.85–17.89)	0.081

Radiotherapy				
No	Reference			
Yes	1.21 (0.59–2.46)	0.608		

HR, hazard ratio; CI, confidence interval.

## Data Availability

The data used in this paper are accessible from the Surveillance, Epidemiology, and End Results (SEER) database on reasonable request at https://seer.cancer.gov/.

## Supplementary Materials

Supplementary Materials
*Supplementary Table 1.* The 5-year and 10-year OS and CSS of the whole cohort, radiotherapy group, and nonradiotherapy group before propensity score matching. *Supplementary Table 2.* The Univariate and multivariate Cox proportional hazard models for overall survival before propensity score matching. *Supplementary Table 3.* The Univariate and multivariate Cox proportional hazard models for cancer-specific survival before propensity score matching. *Supplementary Table 4.* The 5-year and 10-year OS and CSS of the whole cohort, radiotherapy group, and nonradiotherapy group after propensity score matching.

## References

[1] Sbaraglia M., Bellan E., Dei Tos A. P. (2020). The 2020 WHO classification of soft tissue tumours: news and perspectives. *Pathologica*.

[2] von Mehren M., Randall R. L., Benjamin R. S. (2018). Soft tissue sarcoma, version 2.2018, NCCN clinical practice guidelines in Oncology. *Journal of the National Comprehensive Cancer Network*.

[3] Vijay A., Ram L. (2015). Retroperitoneal liposarcoma: a comprehensive review. *American Journal of Clinical Oncology*.

[4] Lu J., Wood D., Ingley E., Koks S., Wong D. (2021). Update on genomic and molecular landscapes of well-differentiated liposarcoma and dedifferentiated liposarcoma. *Molecular Biology Reports*.

[5] Keung E. Z., Hornick J. L., Bertagnolli M. M., Baldini E. H., Raut C. P. (2014). Predictors of outcomes in patients with primary retroperitoneal dedifferentiated liposarcoma undergoing surgery. *Journal of the American College of Surgeons*.

[6] Ghadimi M. P., Al-Zaid T., Madewell J. (2011). Diagnosis, management, and outcome of patients with dedifferentiated liposarcoma systemic metastasis. *Annals Surgical Oncology*.

[7] Bill K. L. J., Garnett J., Meaux I. (2016). SAR405838: a novel and potent inhibitor of the MDM2:p53 Axis for the treatment of dedifferentiated liposarcoma. *Clinical Cancer Research*.

[8] Wu J., Qian S., Jin L. (2019). Prognostic factors of patients with extremity myxoid liposarcomas after surgery. *Journal of Orthopaedic Surgery and Research*.

[9] Cassier P. A., Kantor G., Bonvalot S. (2014). Adjuvant radiotherapy for extremity and trunk wall atypical lipomatous tumor/well-differentiated LPS (ALT/WD-LPS): a French Sarcoma Group (GSF-GETO) study. *Annals of Oncology*.

[10] Haas R. L. M., Bonvalot S., Miceli R. (2019). Radiotherapy for retroperitoneal liposarcoma: a report from the transatlantic retroperitoneal sarcoma working group. *Cancer*.

[11] Callegaro D., Miceli R., Bonvalot S. (2018). Impact of perioperative chemotherapy and radiotherapy in patients with primary extremity soft tissue sarcoma: retrospective analysis across major histological subtypes and major reference centres. *European Journal of Cancer*.

[12] Roeder F., de Paoli A., Saleh-Ebrahimi L. (2018). Intraoperative electron radiation therapy combined with external beam radiation therapy after gross total resection in extremity soft tissue sarcoma: a European pooled analysis. *Ann Surg Oncol*.

[13] Roeder F., Alldinger I., Uhl M. (2018). Intraoperative electron radiation therapy in retroperitoneal sarcoma. *International Journal of Radiation Oncology × Biology × Physics*.

[14] Trovik L. H., Ovrebo K., Almquist M. (2014). Adjuvant radiotherapy in retroperitoneal sarcomas. A Scandinavian Sarcoma Group study of 97 patients. *Acta Oncologica*.

[15] Wang S., Zhou Y., Wang H., Ling J. (2021). Survival analysis and treatment strategies for limb liposarcoma patients with metastasis at presentation. *Medicine (Baltimore)*.

[16] Ye L., Hu C., Wang C., Yu W., Liu F., Chen Z. (2020). Nomogram for predicting the overall survival and cancer-specific survival of patients with extremity liposarcoma: a population-based study. *BMC Cancer*.

[17] Greto D., Saieva C., Loi M. (2019). Influence of age and subtype in outcome of operable liposarcoma. *Radiol Med*.

[18] Toulmonde M., Bonvalot S., Ray-Coquard I. (2014). Retroperitoneal sarcomas: patterns of care in advanced stages, prognostic factors and focus on main histological subtypes: a multicenter analysis of the French Sarcoma Group. *Annals of Oncology*.

[19] Smith C. A., Martinez S. R., Tseng W. H. (2012). Predicting survival for well-differentiated liposarcoma: the importance of tumor location. *Journal of Surgical Research*.

[20] Keung E. Z., Ikoma N., Benjamin R., Wang W. L., Lazar A. J., Feig B. W. (2018). The clinical behavior of well differentiated liposarcoma can be extremely variable: a retrospective cohort study at a major sarcoma center. *J Surg Oncol*.

[21] Dalal K. M., Antonescu C. R., Singer S. (2008). Diagnosis and management of lipomatous tumors. *Journal of Surgical Oncology*.

