# Supplementary material for: Evaluation of Efficacy of Adjuvant Radiotherapy in Well-Differentiated Liposarcoma Patients with Positive Surgical Margins: A Population-based Study
Source: J Oncol. 2022 Sep 8;2022:5735679. doi: 10.1155/2022/5735679 (PMC9477581; doi:10.1155/2022/5735679)
Supplement: Supplementary Materials — Supplementary Table 1. The 5-year and 10-year OS and CSS of the whole cohort, radiotherapy group, and nonradiotherapy group before propensity score matching. Supplementary Table 2. The Univariate and multivariate Cox proportional hazard models for overall survival before propensity score matching. Supplementary Table 3. The Univariate and multivariate Cox proportional hazard models for cancer-specific survival before propensity score matching. Supplementary Table 4. The 5-year and 10-year OS and CSS of the whole cohort, radiotherapy group, and nonradiotherapy group after propensity score matching. [file 5735679.f1.docx]

Supplementary Table 1. The 5-year and 10-year OS and CSS of the whole cohort, radiotherapy group, and non-radiotherapy group **before propensity score matching**.

|  | Overall (95% CI, %) | Radiotherapy group (95% CI, %) | Non-radiotherapy group (95% CI, %) | P value |
| --- | --- | --- | --- | --- |
| 5-year OS | 90.11 (88.71-91.35) | 89.27 (85.42-92.15) | 90.30 (88.77-91.63) | 0.578 |
| 10-year OS | 77.98 (75.72-80.06) | 76.84 (71.11-81.59) | 78.24 (75.75-80.52) | 0.632 |
| 5-year CSS | 96.26 (95.33-97.01) | 95.43 (92.52-97.22) | 96.44 (95.43-97.23) | 0.418 |
| 10-year CSS | 92.86 (91.36-94.11) | 92.82 (88.87-95.41) | 92.85 (91.18-94.22) | 0.987 |

OS, overall survival. CSS, cancer-specific survival.

Supplementary Table 2. Univariate and multivariate Cox proportional hazard models for overall survival **before propensity score matching.**

| Variable | Univariate analysis | | Multivariate analysis | |
| --- | --- | --- | --- | --- |
|  | HR (95% CI) | P value | HR (95% CI) | P value |
| Age |  |  |  |  |
| <=60y | Reference |  | Reference |  |
| >61y | 3.71 (3.00-4.59) | <0.001 | 3.73 (3.02-4.63) | <0.001 |
| Sex |  |  |  |  |
| Male | Reference |  | Reference |  |
| Female | 0.64 (0.53-0.78) | <0.001 | 0.62 (0.51-0.76) | <0.001 |
| Year of diagnosis |  |  |  |  |
| 2000-2009 | Reference |  |  |  |
| 2010-2018 | 0.89 (0.70-1.12) | 0.321 |  |  |
| Race |  |  |  |  |
| White | Reference |  |  |  |
| Black | 0.84 (0.59-1.19) | 0.325 |  |  |
| Other | 1.02 (0.75-1.39) | 0.902 |  |  |
| Primary site |  |  |  |  |
| Retroperitoneum | Reference |  | Reference |  |
| Head and neck | 0.69 (0.41-1.18) | 0.175 | 0.73 (0.43-1.25) | 0.258 |
| Trunk and extremities | 0.42 (0.33-0.54) | <0.001 | 0.44 (0.34-0.56) | <0.001 |
| Visceral organs | 0.75 (0.58-0.96) | 0.025 | 0.69 (0.53-0.89) | 0.004 |
| Tumor size |  |  |  |  |
| <5 cm | Reference |  |  |  |
| 5-10 cm | 0.94 (0.70-1.27) | 0.696 |  |  |
| >=10 cm | 1.13 (0.85-1.51) | 0.388 |  |  |
| Chemotherapy |  |  |  |  |
| No | Reference |  | Reference |  |
| Yes | 4.28 (2.51-7.29) | <0.001 | 4.07 (2.37-7.01) | <0.001 |
| Radiotherapy |  |  |  |  |
| No | Reference |  |  |  |
| Yes | 0.93 (0.72-1.19) | 0.549 |  |  |

HR, hazard ratio; CI, confidence interval.

Supplementary Table 3. Univariate and multivariate Cox proportional hazard models for cancer-specific survival **before propensity score matching.**

| Variable | Univariate analysis | | Multivariate analysis | |
| --- | --- | --- | --- | --- |
|  | HR (95% CI) | P value | HR (95% CI) | P value |
| Age |  |  |  |  |
| <=60y | Reference |  | Reference |  |
| >61y | 1.88 (1.33-2.65) | <0.001 | 1.85 (1.30-2.62) | 0.001 |
| Sex |  |  |  |  |
| Male | Reference |  | Reference |  |
| Female | 0.68 (0.47-0.97) | 0.032 | 0.58 (0.40-0.84) | 0.004 |
| Year of diagnosis |  |  |  |  |
| 2000-2009 | Reference |  |  |  |
| 2010-2018 | 0.96 (0.64-1.44) | 0.857 |  |  |
| Race |  |  |  |  |
| White | Reference |  | Reference |  |
| Black | 0.68 (0.33-1.40) | 0.299 | 0.99 (0.48-2.05) | 0.984 |
| Other | 1.68 (1.06-2.67) | 0.026 | 1.70 (1.07-2.70) | 0.024 |
| Primary site |  |  |  |  |
| Retroperitoneum | Reference |  | Reference |  |
| Head and neck | 0.09 (0.01-0.62) | 0.015 | 0.14 (0.02-1.06) | 0.057 |
| Trunk and extremities | 0.12 (0.07-0.19) | <0.001 | 0.13 (0.08-0.21) | <0.001 |
| Visceral organs | 0.55 (0.38-0.80) | 0.002 | 0.55 (0.37-0.81) | 0.002 |
| Tumor size |  |  |  |  |
| <5 cm | Reference |  | Reference |  |
| 5-10 cm | 1.20 (0.60-2.42) | 0.602 | 1.03 (0.51-2.09) | 0.936 |
| >=10 cm | 2.53 (1.31-4.86) | 0.005 | 2.12 (1.09-4.13) | 0.028 |
| Chemotherapy |  |  |  |  |
| No | Reference |  | Reference |  |
| Yes | 11.75 (6.48-21.29) | <0.001 | 6.65 (3.61-12.27) | <0.001 |
| Radiotherapy |  |  |  |  |
| No | Reference |  |  |  |
| Yes | 0.95 (0.60-1.48) | 0.811 |  |  |

HR, hazard ratio; CI, confidence interval.

Supplementary Table 4. The 5-year and 10-year OS and CSS of the whole cohort, radiotherapy group, and non-radiotherapy group **after propensity score matching**.

|  | Overall (95% CI, %) | Radiotherapy group (95% CI, %) | Non-radiotherapy group (95% CI, %) | P value |
| --- | --- | --- | --- | --- |
| 5-year OS | 89.42 (86.53-91.73) | 89.49 (85.65-92.35) | 89.61 (84.70-93.00) | 0.964 |
| 10-year OS | 77.80 (73.64-81.39) | 76.34 (70.48-81.19) | 79.00 (72.55-84.10) | 0.507 |
| 5-year CSS | 96.32 (84.37-97.60) | 95.71 (92.86-97.45) | 97.62 (94.91-98.90) | 0.192 |
| 10-year CSS | 93.59 (90.88-95.51) | 93.09 (89.15-95.63) | 94.50 (90.17-96.95) | 0.540 |

OS, overall survival. CSS, cancer-specific survival.
